# Utilization of phosphate rock as a sole source of phosphorus for uranium biomineralization mediated by *Penicillium funiculosum*

**DOI:** 10.1039/c8ra01344f

**Published:** 2018-04-10

**Authors:** Nan Hu, Ke Li, Yang Sui, Dexin Ding, Zhongran Dai, Dianxin Li, Nieying Wang, Hui Zhang

**Affiliations:** Key Discipline Laboratory for National Defense for Biotechnology in Uranium Mining and Hydrometallurgy, University of South China Hengyang 421001 China; Hunan Taohuajiang Nuclear Power Co., Ltd Yiyang China 413000; Hunan Province Key Laboratory of Green Development Technology for Extremely Low Grade Uranium Resources Hengyang 421001 China dingdxzzz@163.com

## Abstract

In this work, uranium(vi) biomineralization by soluble *ortho*-phosphate from decomposition of the phosphate rock powder, a cheap and readily available material, was studied in detail. *Penicillium funiculosum* was effective in solubilizing P from the phosphate rock powder, and the highest concentration of the dissolved phosphate reached 220 mg L^−1^ (pH = 6). A yellow precipitate was immediately formed when solutions with different concentrations of uranium were treated with PO_4_^3−^-containing fermentation broth, and the precipitate was identified as chernikovite by Fourier transform infrared spectroscopy, scanning electron microscope, and X-ray powder diffraction. Our study showed that the concentrations of uranium in solutions can be decreased to the level lower than maximum contaminant limit for water (50 μg L^−1^) by the Environmental Protection Agency of China when *Penicillium funiculosum* was incubated for 22 days in the broth containing 5 g L^−1^ phosphate rock powder.

## Abstract 2

In this work, uranium(vi) biomineralization by soluble *ortho*-phosphate from decomposition of the phosphate rock powder, a cheap and readily available material, was studied in detail.

## Graphical abstract

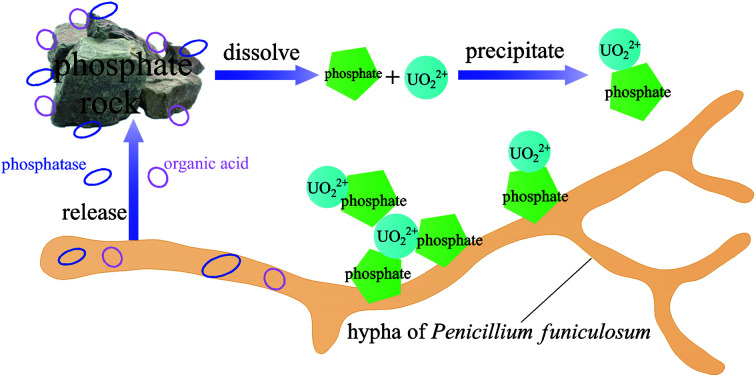

## Introduction

Uranium-containing wastewaters were produced in the process of uranium mining, uranium processing, uranium hydrometallurgy, and nuclear facilities operation.^1^ Due to the long radioactive half-life of uranium, uranium wastewaters can cause serious environmental problems and damage the liver and kidney of human.^2^ The China National Environmental Protection Agency stipulates that the concentration of uranium in the charged wastewater must be below 50 μg L^−1^ (GB 23727-2009), and the U.S. Environmental Protection Agency requires that the concentration of uranium in drinking water should be below 20 μg L^−1^.^3,4^ Therefore, remediation of uranium wastewaters has become the focus of research in environmental problems.

Although many methods (chemical precipitation, liquid–liquid extraction, evaporation, membrane techniques, and ion exchange^5,6^) have been proposed for the decontamination of uranium wastewaters, it remains a challenging work to obtain an effective and economic method for the remediation of uranium wastewaters because many defects exist in the traditional methods, including complicated operation, high cost, secondary contamination and so on.^7^ Alternatively, using microorganism to remediate uranium contaminated water *in situ* has been proved to be a feasible method, especially for the large area and low concentration of uranium wastewater.^8–11^

There are four kinds of mechanisms for the remediation of uranium wastewaters by microorganism: (1) the reduction of aqueous U(vi) into insoluble U(iv) by bacteria (Nitrate-reducing bacteria, sulfate-reducing bacteria, iron-reducing bacteria) in anaerobic reduction environment; (2) the uranyl precipitation due to the interaction of aqueous U(vi) with ligands (SO_4_^2−^, PO_4_^3−^, CO_3_^2−^, OH^−^) released by microbial metabolism products; (3) the adsorption of positively charged uranyl ions onto the negatively charged functional groups on the surface of microbial cells; (4) the entrance of uranium into the cells due to the increase of the permeability of the cells by uranium toxicity.^12^

Uranium usually exists in form of soluble hexavalent uranyl ions (UO_2_^2+^) under the natural oxidation conditions.^13^ The concentration of U(vi) in solution can be decreased by reducing U(vi) to U(iv). However, the U(iv) can be reoxidized into soluble U(vi) when exposed to oxidation environment, and the uranium pollution will be restored due to the reoxidation of U(iv) to U(vi).^14,15^ Therefore, the method of transformation of U into stable uranyl phosphate by the interaction of U(vi) with PO_4_^3−^ is a good strategy for remediation of uranium wastewaters because uranyl phosphate cannot be reoxidized.^16,17^

In recent years, many studies demonstrated that uranium biomineralization was an effective method for remediation of uranium wastewaters.^8,18–21^ For example, Lang's group used glycerol phosphate as the source of phosphorus for uranium biomineralization by yeasts.^18^ Generally, the reported sources of phosphorus were organophosphorus, including glycerol phosphates, phytic acid, tributylphosphate and so on.^12^ But although the phosphate rock powder is cheap and readily available compared to the organophosphorus, investigators have seldom used phosphate rock powder as a source of phosphorus to mineralize uranium, and the reason for this is that microorganisms produce organic acids and acidic phosphatase to dissolve phosphate rock powder, which decreases the value of pH and hinders the formation of uranium precipitation.^22,23^ However, it was recently reported that some microorganisms, such as *Aspergillus awamori*, *Aspergillus oryzae*, *Penicillium oxalicum*, and *Neurospora crassa*, could cause the value of pH to increase during the later stage of fermentation.^24–26^ Therefore, we explored the process of uranium biomineralization by *Penicillium funiculosum* incubated in the broth with phosphate rock powder. In addition, the chemical composition of precipitate of uranium mineral was also inspected.

## Experimental

### Materials and methods

The *Penicillium funiculosum* was isolated from the Guangdong uranium mine tailings mud and identified by Guangdong Institute of Microbiology. Cold storage strains (−80 °C) were inoculated to Potato Dextrose Agar (PDA) at a slab, and the slab was then incubated at 30 °C in a constant temperature incubator for 96 h. The temperature of pressure vapour sterilizer was set at 121 °C during the sterilization processes. The phosphate rock which contains 13% phosphorus was ground as powder (150 mesh).

For preparation of the stock uranium(vi) solution (1 g L^−1^), 10 mL hydrochloric acid (*ρ* = 1.18 g mL^−1^) and 2 mL hydrogen peroxide (30%) were added to a 100 mL beaker containing 1.1792 g of U_3_O_8_, the solution was then heated until it was nearly boiled away and 10 mL hydrochloric acid (*ρ* = 1.18 g mL^−1^) was added, uranium(vi) stock solution was finally obtained by transferring the solution to a 1000 mL volumetric flask and diluting to the mark with deionised water. Other concentrations of uranium solutions were prepared by diluting the stock solution.^27^

The concentration of uranium in solution was measured by inductively coupled plasma mass spectrometer (ICP-MS, Agilent 7700a Series); the concentration of inorganic phosphate in solution was measured by UV-Vis spectrophotometer (phosphorus molybdenum blue spectrophotometry, T6 the new century, Beijing); and the value of pH was measured with pH meter (PHSJ-3F, Chinese Rex). Analytical instruments for sediment included transform infrared spectrometer (iS10, Nicolet), scanning electron microscope with energy dispersive spectrometer (S-4800, Japan Hitachi), and X-ray powder diffractometers (X'Pert pro, Holland PANalytical B.V.).

### Experimental design

#### Dissolving phosphorus experiment

200 mL modified Pikovskaya's broth (consisting of 10 g glucose, 0.5 g KNO_3_, 0.3 g NaCl, 0.3 g MgSO_4_·7H_2_O, 0.03 g FeSO_4_, 0.03 g MnSO_4_·H_2_O, 0.3 g KCl, and 5 g phosphate rock powder^28,29^) was added to 500 mL conical flask, and the pH value was adjusted to 6 with saturated Na_2_CO_3_ solution and 1 mol L^−1^ HCl solution. The broth was sterilized at 121 °C for 30 min before experiments. The experiments were divided into experimental and control groups, and each group included three parallel samples. The experimental group was incubated with 2 mL spore suspension with OD_600_ value of 0.2 obtained from the activated *Penicillium funiculosum*, and the control group was incubated with 2 mL sterilized spore suspension. The experimental and control groups were incubated in a shaker at 30 °C and 140 rpm. 3 mL fermentation broth was taken from the conical flask at proper time and was centrifuged at 8000 rpm for 10 min, and the supernatants were taken for detecting pH and concentration of phosphorus. Finally, the precipitate formed in the experiment was washed eight times with deionised water for next analysis.

#### Biomineralization experiment

200 mL modified Pikovskaya's broth of uranium solution (25 mg L^−1^) was added to a 500 mL conical flask, and the pH value was adjusted to 6 with saturated Na_2_CO_3_ solution and 1 mol L^−1^ HCl solution. The broth was sterilized at 121 °C for 30 min before experiments. The experiments were divided into experimental and control groups, and each group included three parallel samples. The experimental group was incubated with 2 mL spore suspension with OD_600_ value of 0.2 obtained from the activated *Penicillium funiculosum*, and the control group was incubated with 2 mL sterilized spore suspension. The experimental and control groups were incubated in a shaker at 30 °C and 140 rpm. 3 mL fermentation broth was taken from the conical flask every other day and was centrifuged at 8000 rpm for 10 min, and the supernatant was taken for detecting pH concentration of uranium, and concentration of phosphorus. Finally, the precipitation was washed eight times with deionised water for next analysis.

#### Mineralization experiment with fermentation broth

In order to study the influence of the released phosphate on the concentration of uranium in biomineralization experiment, we used the fermentation broth from dissolving phosphorus experiment with *Penicillium funiculosum* to precipitate uranium.^30^ The fermentation broth was passed through a 0.22 μm pore size filter to remove the hypha and phosphate rock powder. 10 mL filtered fermentation broth was added to 50 mL conical flasks containing different concentrations of uranium (25 mg L^−1^, 50 mg L^−1^, 250 mg L^−1^, 500 mg L^−1^, 750 mg L^−1^, and 1 g L^−1^), which were incubated in a shaker at 30 °C and 140 rpm. The solutions were centrifuged at 8000 rpm for 10 min, and the supernatants were taken for detecting concentrations of uranium and dissolved phosphorus after 12 h, and the precipitation was washed eight times with deionised water for next analysis.

## Results and discussion

### Phosphate rock powder solubilization by *Penicillium funiculosum*

**Fig. 1:**
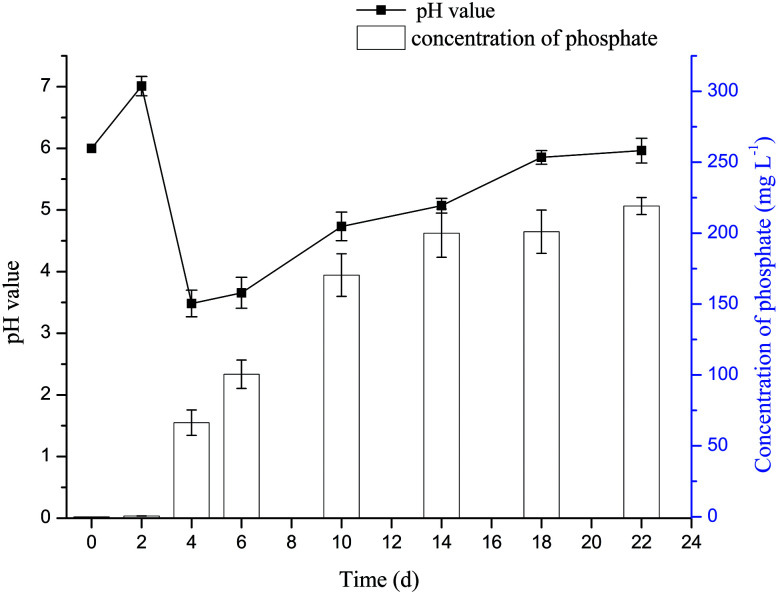
Relationship between the pH value or the concentration of phosphate and the time.

The variation of concentration of phosphate and pH value in the culture medium of dissolving phosphorus experiment with time was shown in Fig. 1. The pH value of the medium was increased from 6 to 7 after the addition of the phosphate rock powder, which may be due to the existed alkaline substances such as calcium carbonate in the phosphate rock powder. Then, the pH value began to decline and reached the lowest level on the fourth day. It was probable that the organic acids, such as glucose acid, oxalic acid and citric acid, produced by microorganisms decreased the pH value.^22^ The pH value increased to about 6 during the later stage (from 4 d to 18 d), which was probably due to the cell autolysis when glucose carbon source was consumed.^24,25^ The variation of pH value in the culture medium during fungal growth was similar to that reported by Amin and Ghazala.^31^ The results showed that phosphate rock powder could be dissolved by organic acid and phosphatase secreted by *Penicillium funiculosum*, giving soluble *ortho*-phosphate (PO_4_^3−^);^32^ and the phosphorus concentration could reach 220 mg L^−1^ after 22 days.

### Uranium biomineralization by *Penicillium funiculosum*

**Fig. 2:**
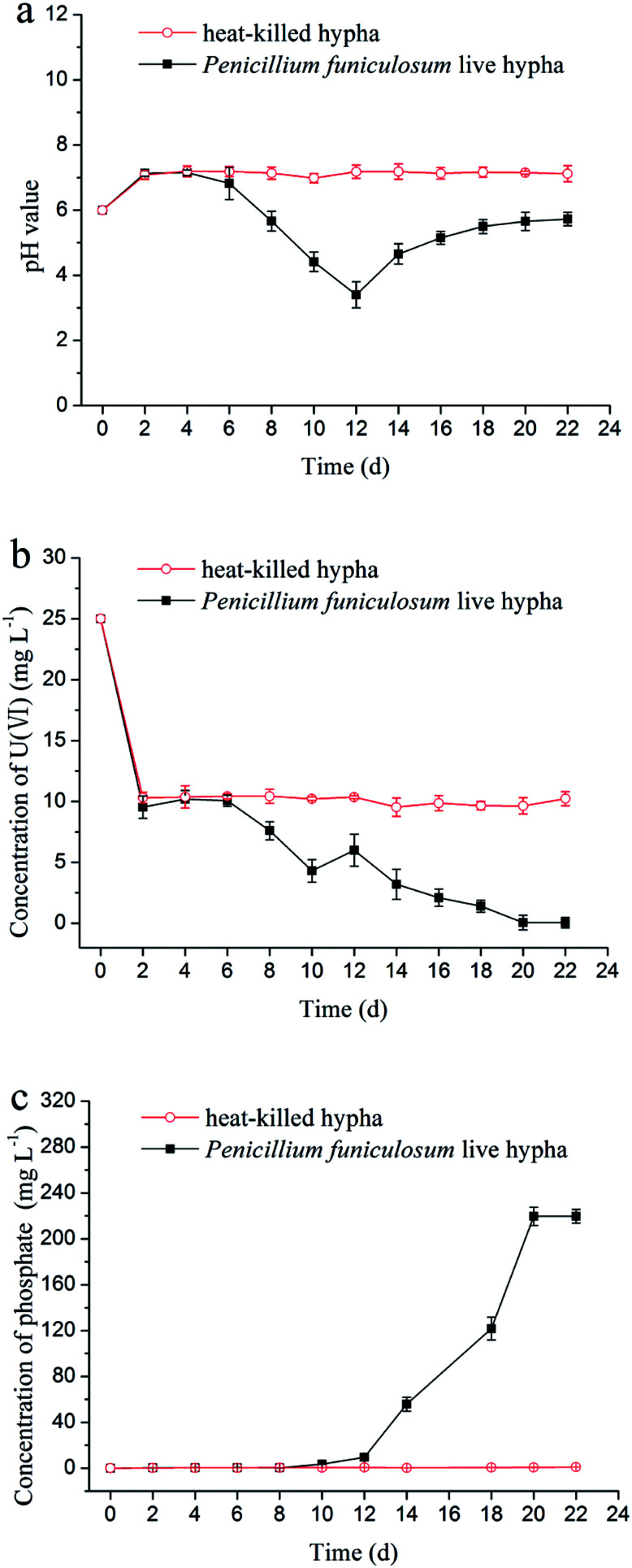
Variations of (a) pH value, (b) concentration of uranium, and (c) concentration of phosphate with time in the uranium biomineralization by *Penicillium funiculosum*.

In the uranium biomineralization experiment, the variation of pH value (Fig. 2a) had a significant delay compared with the dissolving phosphorus experiment, which indicated that uranium would inhibit the growth of *Penicillium funiculosum*.^33^ The value of pH decreased to 3 at day 12, then increased to 6 at day 22, and finally remained stable, which was probably due to the cell autolysis when glucose carbon source was consumed.^24^ On the other hand, the pH value of control group remained unchanged.

The variation of concentration of uranium(vi) is shown in Fig. 2b. First, the uranium concentration decreased from 25 mg L^−1^ to 10 mg L^−1^ in the first two days for experimental group and control group, which was due to the adsorption of phosphate rock powder and microorganism hypha.^34–36^ Then, the concentration of uranium remained unchanged in control group, and the uranium concentration and the pH value in the experimental group began to decline at day 6, which was due to the interaction of uranium with PO_4_^3−^ from the decomposition of phosphate rock powder.^12^ The concentration of uranium was on rise from day 10 to day 12 (Fig. 2b), which was attributable to the too low pH value (less than 4).^23,37,38^ After 12 days, uranium concentration continuously decreased, which was ascribed to the rise of pH value and PO_4_^3−^ concentration, as shown in Fig. 2a and c. Particularly, the uranium concentration decreased to 47.3 μg L^−1^ at day 22 and remained stable afterwards, which was lower than the maximum contaminant limit for the charged wastewater (50 μg L^−1^) by The Environmental Protection Agency of China.

### Mineralization experiment with fermentation broth

**Fig. 3:**
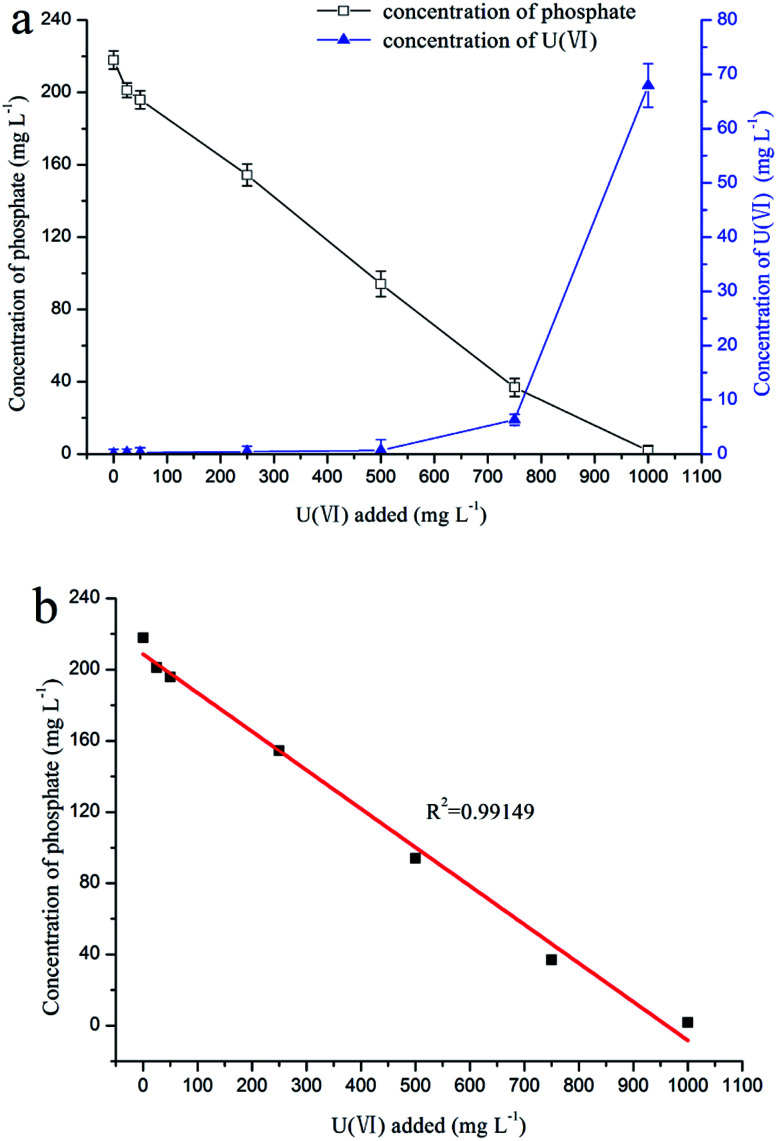
Relationships (a) between the concentration of phosphate or uranium and the concentration of the added U(vi), and (b) between the concentration of phosphate and the concentration of the added U(vi).

The results of the mineralization experiment with fermentation broth is shown in Fig. 3a, where the uranium concentration decreased from 25, 50, 250, and 500 mg L^−1^ to 0.2, 0.3, 0.4, and 0.6 mg L^−1^, respectively, and the concentration of phosphorus decreased from 220 mg L^−1^ to 201.2, 195.9, 154.4, and 94.1 mg L^−1^, respectively. Obviously, the phosphorus concentration was positively correlated with the uranium concentration (Fig. 3b, *R*^2^ = 0.99149). The results indicate that uranyl phosphate solid may form by the reaction of uranium with phosphorus.

### FT-IR spectroscopy analysis

**Fig. 4:**
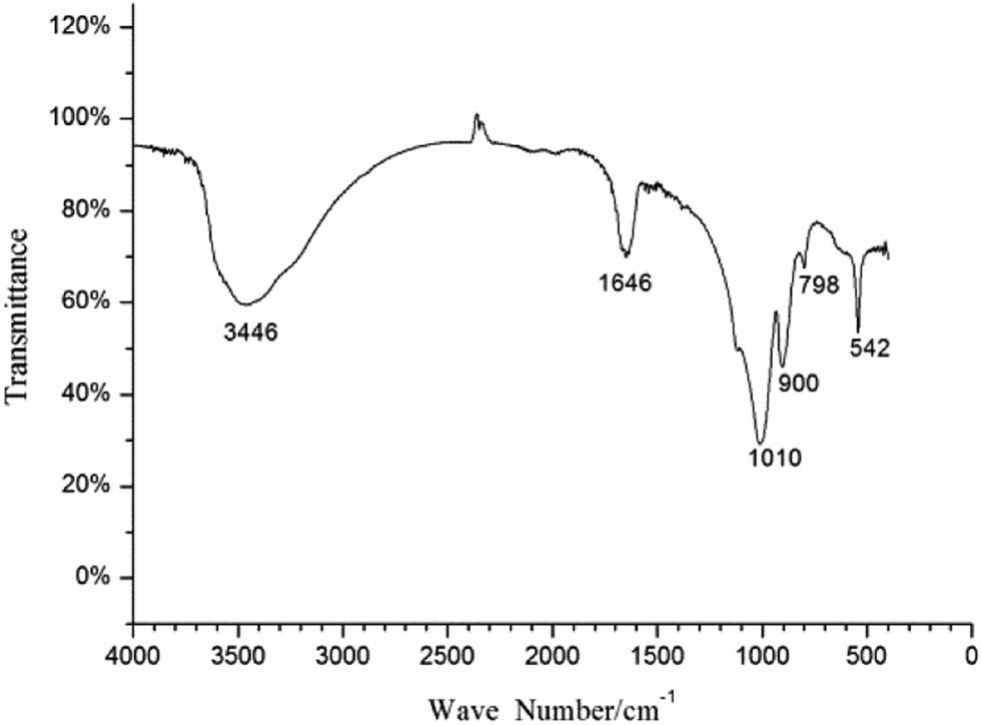
FT-IR spectra of the precipitate formed in the mineralization experiment with fermentation broth.

The precipitate generated in the mineralization experiment with fermentation broth was analyzed by FT-IR, and the result is shown in Fig. 4. The peaks at 1646 cm^−1^ and 3446 cm^−1^ may correspond to the bending vibration and antisymmetric stretching vibration of OH^−^. The peak at 542 cm^−1^ can be assigned to bending vibration of PO_4_^3−^; the peaks at 798 cm^−1^ and 900 cm^−1^ may correspond to the symmetric stretching vibration and antisymmetric stretching vibration of UO_2_^2+^, respectively; and the peak at 1010 cm^−1^ may be related to antisymmetric stretching vibration of PO_4_^3−^.^39,40^ The FT-IR analysis indicates that PO_4_^3−^ played an important role in the formation of uranium precipitate; and the precipitate was U–P mineral. The results of FT-IR matched well with the studies by Armstrong *et al.* and Clavier *et al.*^41,42^

### SEM and EDS results

**Fig. 5:**
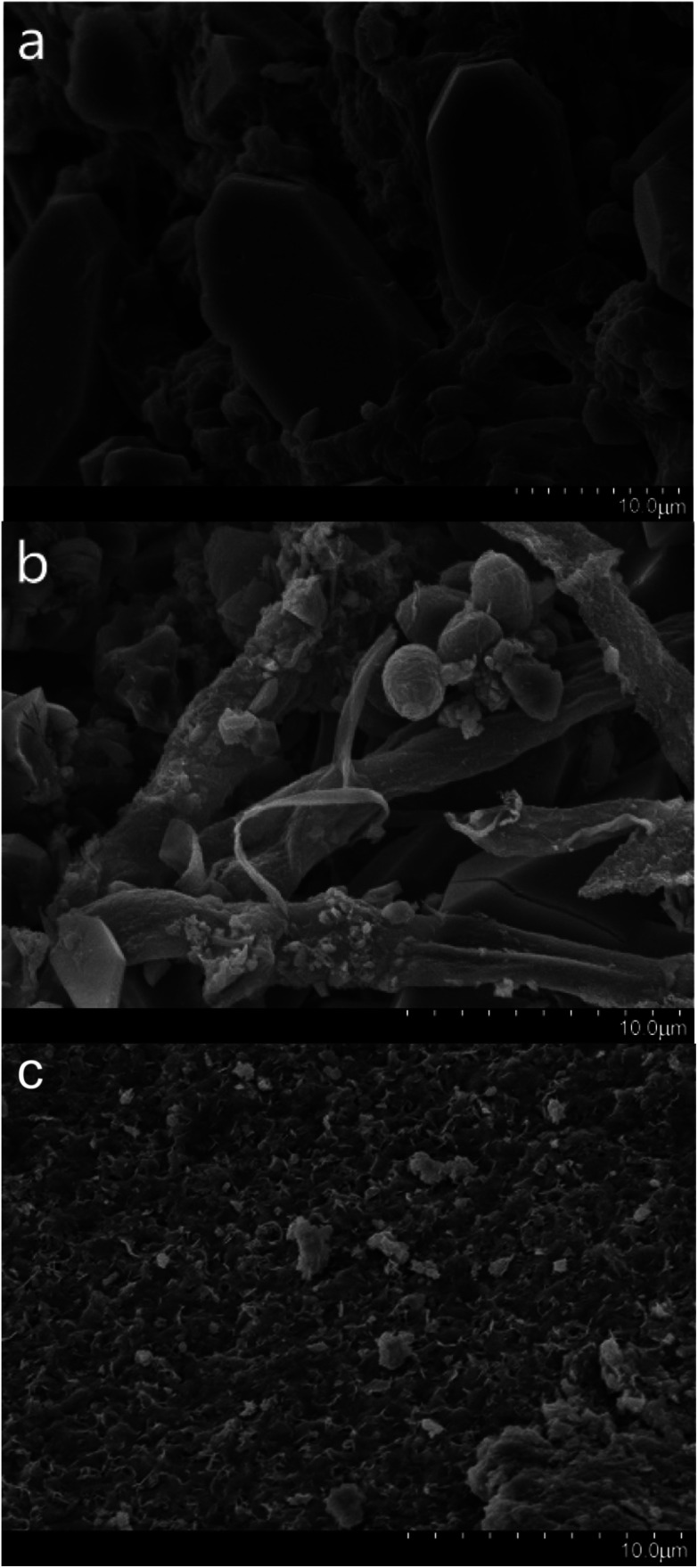
SEM images of precipitates formed in (a) dissolving phosphorus experiment (scale bar: 10 μm), (b) biomineralization experiment (scale bar: 10 μm), and (c) mineralization experiment with fermentation broth (scale bar: 10 μm).

**Fig. 6:**
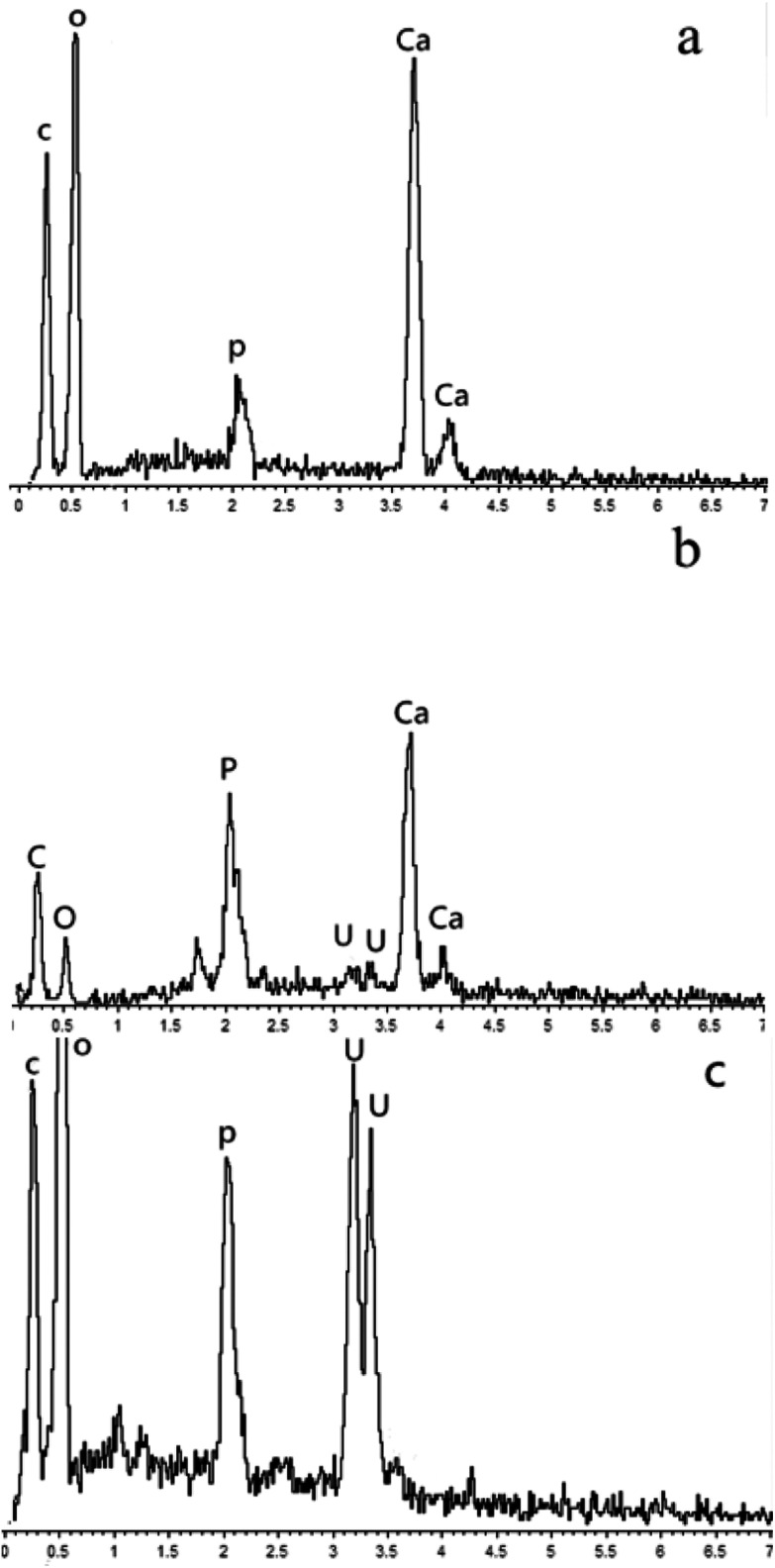
EDS images of precipitates formed in (a) dissoluting phosphours experiment (b) biomineralization experiment and (c) mineralization experiment with fermentation broth.

SEM was used for the analysis of obtained precipitates from the dissolving phosphorus experiment, biomineralization experiment, and mineralization experiment with fermentation broth, and the results are shown in Fig. 5. Prismatic crystals and mycelium were found in the precipitates from the dissolving phosphorus experiment and biomineralization experiment (Fig. 5a and b). The sediment could adhere onto the mycelium surface in Fig. 5b, which showed that uranium mineralization could occurred on the surface of hyphae.^43^ The SEM image of U–P mineral was given in Fig. 5c, and the disappearance of prismatic crystals and mycelium may be due to the filtration. In the energy-dispersive spectrum (EDS), peaks of phosphorus and uranium in the biomineralization experiment and mineralization experiment with fermentation broth further indicated that the U–P mineral was formed (Fig. 6b and c).^44^

### XRD results

**Fig. 7:**
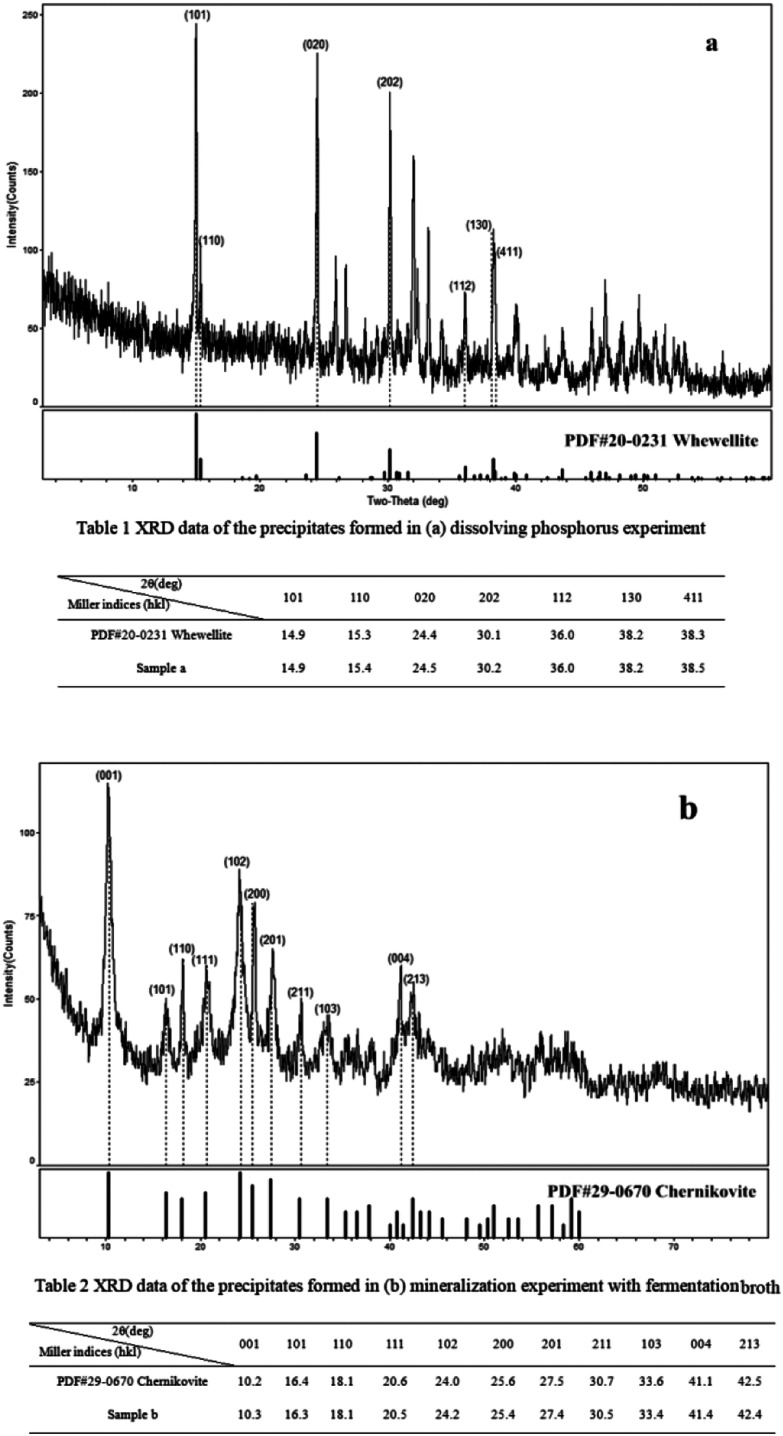
XRD images of the precipitates formed in (a) dissolving phosphorus experiment, and (b) mineralization experiment with fermentation broth.

The X-ray Diffraction (XRD) patterns of the precipitate formed in the dissolving phosphorus experiment is shown in Fig. 7a and Table 1. The matching of the three highest reflections with the standard photographs of certified PDF 20-0231 for calcium oxalate indicates that the prismatic crystals found in scanning electron micrographs (Fig. 5a) was most likely the calcium oxalate.^45^ This proved that the calcium ions released from the process of dissolving phosphate rock powder by *Penicillium funiculosum* formed the insoluble calcium oxalate mineral. In order to ascertain the type of uranium mineral, the precipitate formed in the mineralization experiment with fermentation broth was analyzed by XRD. The results are shown in Fig. 7b and Table 2, and its map matched with reference patterns for chernikovite (PDF 29-0670 H_2_ (UO_2_)_2_(PO_4_)_2_·8H_2_O), confirming that the uranium existed in the form of uranyl phosphate minerals.^30^ The U(vi) in the solution could be biomineralized by microbes and plants to form chernikovite, which has also been confirmed by other researchers.^46–49^

## Conclusions

The phosphate rock powder was successfully used as a sole source of phosphorus for the uranium biomineralization. *Penicillium funiculosum* was effective in dissolving phosphate rock powder and yielding PO_4_^3−^, and the phosphorus concentration in fermentation broth could reach 220 mg L^−1^ when it was incubated for 22 days. The uranium concentration in solution could be decreased from 25 mg L^−1^ to 47.3 μg L^−1^ after 22 days of incubation. The FT-IR, SEM and EDS analyses showed that the precipitated uranium was a U–P mineral, and the XRD analysis further confirmed that the mineral was a uranyl phosphate mineral.

## Conflicts of interest

There are no conflicts to declare.

## Supplementary Material
